# Effect of Temperature on Xylanase II from *Trichoderma reesei* QM 9414: A Calorimetric, Catalytic, and Conformational Study

**DOI:** 10.1155/2014/708676

**Published:** 2014-09-07

**Authors:** Gloria López, Pilar Estrada

**Affiliations:** Departamento de Bioquímica y Biología Molecular I, Facultad de Biología, Universidad Complutense, 28040 Madrid, Spain

## Abstract

The secondary structure of xylanase II from* Trichoderma reesei* is lost in an apparent irreversible cooperative process as temperature is increased with a midpoint transition of 58.8 ± 0.1°C. The shift of the spectral centre of mass above 50°C is also apparently cooperative with midpoint transition of 56.3 ± 0.2°C, but the existence of two isofluorescent points in the fluorescence emission spectra suggests a non-two-state process. Further corroboration comes from differential scanning calorimetry experiments. At protein concentrations ≤0.56 mg*·*mL^−1^ the calorimetric transition is reversible and the data were fitted to a non-two-state model and deconvoluted into six transitions, whereas at concentrations greater than 0.56 mg*·*mL^−1^ the calorimetric transition is irreversible with an exothermic contribution to the thermogram. The apparent *T*_*m*_ increased linearly with the scan rate according to first order inactivation kinetics. The effect of additives on the calorimetric transition of xylanase is dependent on their nature. The addition of sorbitol transforms reversible transitions into irreversible transitions while stabilizing the protein as the apparent *T*_*m*_ increases linearly with sorbitol concentration. d-Glucono-1,5-lactone, a noncompetitive inhibitor in xylanase kinetics, and soluble xylan change irreversible processes into reversible processes at high protein concentration.

## 1. Introduction

Xylan is the most abundant polysaccharide after cellulose [1]. The filamentous fungus* Trichoderma reesei* (anamorph of* Hypocrea jecorina*) produces xylanolytic enzymes to hydrolyze xylan. One of them, xylanase or 1,4-*β*-D-xylan xylanohydrolase (EC 3.2.1.8), hydrolyzes the *β*-1,4-bonds in the main chain generating a mixture of xylooligosaccharides [2]. The high activity of some xylanases makes them very attractive for industrial purposes. Xylanases are used to reduce the harsh chemicals in the chemical bleaching stages of kraft pulp by improving the lignin release through xylan hydrolysis [3]. In textile industry, xylanases are an alternative to the sulphuric acid treatment of the textile polyester-cellulose wastes [4] and in the food industry xylanases improve the dough properties and baking quality of goods by breaking down the polysaccharides in the dough [5]. As several industrial processes employ high temperatures and many xylanases are obtained from mesophilic microorganisms, the improvement of xylanases thermostability is a challenge nowadays. To achieve it, a considerable lot of work has been invested in past years. Among the strategies followed to increase the thermal stability of the enzyme, a favourite has been the introduction of disulfide bond by means of site-directed mutagenesis [6, 7] as well as the introduction of arginines [8]. Addition of seven thermophilic derived residues onto a mesophilic xylanase was sufficient to confer thermostability to this engineered variant [9]. Other attempts of improving xylanase thermostability have been only partially successful since the mutants were thermostable compared with the wild type but less active at low temperatures [10, 11]. Shuffling between a mesophilic and a thermophilic homologue has been also assayed in order to increase the thermal stability of mesophilic enzyme without cost to its activity [12]. Paralleling the mentioned studies, other works have dealt with the characterization of the temperature-dependent inactivation process of xylanases by using techniques such as electrospray ionization Fourier-transform ion cyclotron-resonance mass-spectrometry and dynamic light scattering [13], differential scanning calorimetry (DSC) [14–18], circular dichroism (CD) [19], and Fourier-transform infrared spectroscopic characterization [20]. Molecular dynamics has also been employed to simulate the thermal unfolding of family 11 xylanases [21]. However, to our knowledge, a thermoinactivation study of xylanases, correlating structural, catalytic, and calorimetric studies while considering the effect that sorbitol (an enzyme stabilizer) and ligands have on xylanase thermoinactivation measured by DSC, has not yet been carried out.

To date, four xylanases have been described in* Trichoderma reesei*: xylanase I (19 kDa, pI ~ 5.2), xylanase II, one of the major xylanases of the fungus (21 kDa, pI ~ 9) [2, 22], xylanase III (32 kDa, pI ~ 9.1), practically absent in* T. reesei* QM 9414 [23], and xylanase IV (50.3 kDa, pI ~ 7) [24, 25]. For several years, we have studied the production of cellulolytic enzymes by the fungus* Trichoderma reesei* QM 9414 grown on wheat straw [26]. As the fungus growing on straw produces also hemicellulases, we have purified xylanase II and studied its stability to chemical denaturants, trifluoroethanol, and pH changes in order to characterize the intermediate states in the unfolding process [27]. The thermal stability of xylanase II has been improved in the presence of polyols, some of them being also inhibitors on the enzyme kinetics [28]. In the present paper, we have studied the effect of temperature on the secondary and tertiary structure of the enzyme by means of CD and fluorescence emission spectroscopies as well as on the catalytic stability and activity of the enzyme. Further information on the effect of temperature on xylanase II alone or in the presence of ligands (soluble xylan and d-glucono-1,5-lactone, GL) and stabilizers (sorbitol) was gained through DSC. The thermal denaturation of xylanase II was reversible or irreversible depending on protein concentration and the presence of additives changed reversible into irreversible transitions (sorbitol), whereas ligands (GL and soluble xylan) change irreversible to reversible ones.

## 2. Materials and Methods

### 2.1. Enzyme Purification and Molecular Characterization

*Trichoderma reesei* QM 9414 was cultured on wheat straw as previously described [26]. Xylanase was purified from supernatant culture with ammonium sulphate precipitation, DEAE-Sepharose CL-6B (Pharmacia, Sweden), and Ultrogel AcA 44 (LKB, Sweden) chromatographies [27, 28]. The fractions corresponding to the top of the elution peak of the last chromatography were pooled, dialyzed towards the needed buffer, and employed for structural, catalytic, or calorimetric studies. The purity of xylanase was routinely checked by SDS-PAGE and occasionally by amino acid analysis. For glycoprotein staining, the gel was treated with 12.5% (w/v) trichloroacetic acid for 30 min, washed with water, treated with 3% (v/v) acetic acid and 1% (w/v) periodic acid for 1 h, washed again, treated with the Schiff reagent for 1 h in darkness, washed first with 0.5% (w/v) sodium metabisulfite for 10 min, and then washed with water.

Hydrodynamic studies of 0.4 mg*·*mL^−1^ in 0.1 M citrate buffer pH 5 preheated at 50°C for 10 min were performed at 25°C and 4,8000 rpm in an Optima XL-I (Beckman-Coulter Inc.) analytical ultracentrifuge equipped with UV-visible optics at the CIB of the CSIC (Madrid). The partial specific volume of xylanase, 0.73 m*·*g^−1^, was estimated from its amino acid composition with the SEDNTERP program. The solvent density was 1.015 g*·*mL^−1^ and the measured solvent viscosity was 0.011018 poise. The standard sedimentation coefficient in water at 20°C, sw_(20,w)_, was calculated from the experimental coefficient taking into account the density and the viscosity at 20°C.

### 2.2. Enzymatic Assays

Enzymatic assays were carried out as previously described [28]. Stability assays under different temperatures were carried out by preincubating 1.3 *μ*g protein for 30 min in standard buffer in a water bath at the indicated temperature. After cooling in ice for 10 min, enzymatic assays were carried out in standard conditions. Enzymatic assays in the presence of 0.5–0.26 M sorbitol (Sigma) or 0.1–0.6 M d-glucono-1,5-lactone (GL, Sigma) were carried out in standard conditions. To obtain beechwood soluble xylan, a suspension of xylan in water (2%, w/v) was autoclaved for 1 h at 120°C (2 kg*·*cm^−1^) and centrifuged at 5,000 rpm for 1 h at 4°C. The supernatant was freeze-dried, weighted, the yield being 92% (w/w), and solved in buffer to reach 5% (w/v).

### 2.3. Analytics

Amino acid analysis of xylanase (0.8–2.3 nmol) was determined on a Biochrom 30 automatic amino acid analyser after hydrolysis with 6 N HCl at 105°C for 24 h in sealed tubes under vacuum. The protein concentration of samples was calculated from their absorbance at 280 nm using the experimental molar absorption coefficient *Є*_280_ = 58453 ± 212 M^−1^cm^−1^ calculated from the amino acid content [27]. Reducing sugars were measured by the dinitrosalicylic reagent method [29] recording the absorbance of the reduced reagent at 530 or 640 nm in a Beckman DU-800 spectrophotometer with d-xylose (Sigma) as standard. The actual GL and d-gluconic acid contents of GL solutions in standard buffer were determined by polarimetry at 20°C in a Perkin Elmer 241 polarimeter as previously described [30]. As mutarotation was achieved in less than one hour, the GL stock solution (3.4 M) was prepared at least two hours before being used, to ensure the same actual mixture of acid and lactone concentrations in it [30].

### 2.4. Circular Dichroism Studies

Far-UV circular dichroism (CD) spectra of xylanase were recorded at 20°C on a Jasco J-715 spectropolarimeter in thermostated quartz cells of 0.1-cm path length, at 50 nm*·*min^−1^ (1 s response time) for the far-UV (240–200 nm) spectral range, each spectrum being the accumulation of 5 scans. The spectra were obtained with 0.09 mg*·*mL^−1^ protein in 200 *μ*L of 20 mM citrate buffer pH 5. Estimations of the secondary structure content from the CD spectra were performed with the CDPro program; the α-helix, *β*-sheet, random, and turn contents were the mean of values calculated using three different methods, CONTIN/LL, SELCON3, and CDSSTR [31]. CD spectra of xylanase with additives were recorded under the same conditions except that 2 M sorbitol, 0.4 M gluconolactone, or 0.5% (w/v) soluble xylan was added to xylanase samples. The temperature dependence of the CD signal was determined by heating the sample from 20 to 85°C at 30°C*·*h^−1^, collecting data every 0.2°C, and recording the ellipticity at 220 nm. After the T ramp, the sample was cooled back to 20°C at 30°C*·*h^−1^ and the ellipticity of the sample at 220 nm was again recorded.

### 2.5. Intrinsic and Extrinsic Fluorescence Spectroscopy Studies

The intrinsic fluorescence emission spectra of xylanase were recorded at 19°C in SLM-Aminco AB2 spectrofluorometer using 1-cm quartz cell with excitation either at 275 nm or at 290 nm. Emission slits were set at 4 nm and scan speed was 2 nm*·*s^−1^. Samples contained 0.09 mg*·*mL^−1^ protein in 20 mM citrate buffer pH 5. The spectrum obtained upon excitation at 290 nm was normalized multiplying it by a factor to determine the contribution of tryptophan residues. This factor is the ratio between the fluorescence intensities at *λ*_exc_ = 275 and *λ*_exc_ = 290 nm at wavelengths higher than 380 nm where no tyrosine contributes to the fluorescence emission [32]. The tyrosine contribution to the emission spectrum was calculated by subtracting from the emission spectrum at *λ*_exc_ = 275 nm the normalized emission spectrum due to tryptophan residues. The fluorescence emission of xylanase with 0.5–2 M sorbitol, 0.2–0.4 M gluconolactone, or 0.5% (w/v) soluble xylan was obtained under the same conditions as described above. The effect of temperature (19°C to 70°C) on the fluorescence emission of xylanase was carried out upon excitation at 290 nm. The emission of N-acetyl-L-tryptophanamide (NATA, Sigma) was recorded under identical conditions to the protein experiment and at the same molar concentration (25.92 *μ*M) of trp residues in xylanase [33].

### 2.6. Differential Scanning Calorimetry

Differential scanning calorimetry (DSC) experiments were performed on a VP-DSC (MicroCal) differential scanning microcalorimeter with a cell volume of 514.9 *μ*L. Xylanase (0.4 to 1.1 mg*·*mL^−1^) was dialyzed towards 0.1 M sodium citrate buffer pH 5 and both sample and buffer were degassed for 3 min at room temperature in a chamber under vacuum and gentle stirring and then loaded into the sample- and reference-cells, respectively, where overpressure was kept to prevent degassing. Measures were taken every 0.1°C and the scan rate was 60°C*·*h^−1^ unless otherwise indicated. DSC scans were terminated several degrees above the end of the calorimetric transition to ensure that the posttransition heat capacity levels are well defined. The second scans were obtained by reheating samples after cooling for 20 min from the first one. The *C*_*p*_ profiles were obtained by subtracting the instrumental baseline (obtained with buffer in both cells) from the experimental thermograms. Then, the thermograms were normalized for protein concentration based on a monomer of 20,842 g*·*mol^−1^ (DNA star program), and then pre- and posttransition baselines were subtracted. Samples of 0.4 mg*·*mL^−1^ protein in 0.1 M sodium citrate buffer pH 5, containing 0.5–2 M sorbitol, prepared from a stock 3.08 M in the same buffer were subjected to DSC at 60°C*·*h^−1^ scan rate. Samples (1 mg*·*mL^−1^ protein) with 0.4 M Gl were prepared from a stock of 3.4 M GL in the same buffer. The scan rate was 15°C*·*h^−1^. Samples containing 0.5 (w/v) soluble xylan from a 5% (w/v) stock were added to 1 mg*·*mL^−1^ protein solution in 0.1 M citrate buffer pH 5 and the transition was carried out at 15°C*·*h^−1^ scan rate. The additives were always added to the reference cell at the same concentration rather than to the sample cell.

### 2.7. Analysis of the Data

The CD, SCM (spectral centre of mass, an intensity-weighted average emission wavelength), or percent residual activity versus temperature data was fitted to the following:

(1) $$\begin{array}{c} Y_{\text{obs}}=Y_{N}+\frac{a}{\left(1+\exp\left(-\left(T-T_{m}\right)/b\right)\right)}, \end{array}$$

where *Y*_obs_ is the observed parameter at each temperature, *Y*_*N*_ is the parameter value at the lowest temperature, *T* (°C) is the current temperature, *T*_*m*_ (°C) is the temperature at which half of the protein is unfolded (the midpoint transition), and *a* and *b* are constants. The fluorescence SCM was calculated according to the following:

(2) $$\begin{array}{c} \text{SCM}=\Sigma \lambda \cdot \frac{I\left(\lambda \right)}{\Sigma I\left(\lambda \right)}, \end{array}$$

where *λ* is the emission wavelength and *I*(*λ*) represents the fluorescence intensity at wavelength *λ*. The apparent activation energy (apEa) of the irreversible denaturation of xylanase by DSC was calculated with the following:

(3) $$\begin{array}{c} \ln\left(\frac{\beta }{{T_{m}}^{2}}\right)=C-\frac{\text{apEa}}{RT_{m}}, \end{array}$$

where *β* is the scan rate (°C*·*h^−1^), *T*_*m*_ (°C) is the apparent melting temperature, and *C* is a constant. The reversible transitions were fitted with the Origin MicroCal software to the non-two-state model with six peaks according to (4) which, in addition to the term represented below, contains 5 more terms (not shown for obvious reasons of length and complexity) as this model accounted for the best fitting of the data (the lowest *χ* ^2^/DoF):

(4) Cp(T) =exp⁡{(−ΔHVH1/RT)(1−T/Tm1)}ΔHVH1ΔHcal1(1+exp⁡{(−ΔHVH1/RT)(1−T/Tm1)})2RT2  +⋯,

where *T*_*m*1_ (°C), the van't Hoff enthalpy change, Δ*H*_VH1_ (kCal*·*mol^−1^), and the calorific enthalpy change, Δ*H*_cal1_ (kCal*·*mol^−1^), account for subdomain 1. The calorimetric data obtained at several protein concentrations are fitted to the following:

(5) $$\begin{array}{c} \ln\left[\text{Prot}\right]=C-\frac{\text{apEa}}{RT_{m}}\frac{\mu }{\left(\mu -1\right)}, \end{array}$$

where *C* is a constant and *μ* is the molecularity of the reaction.

## 3. Results and Discussion

### 3.1. Molecular Characterization of Xylanase

**Figure 1:**
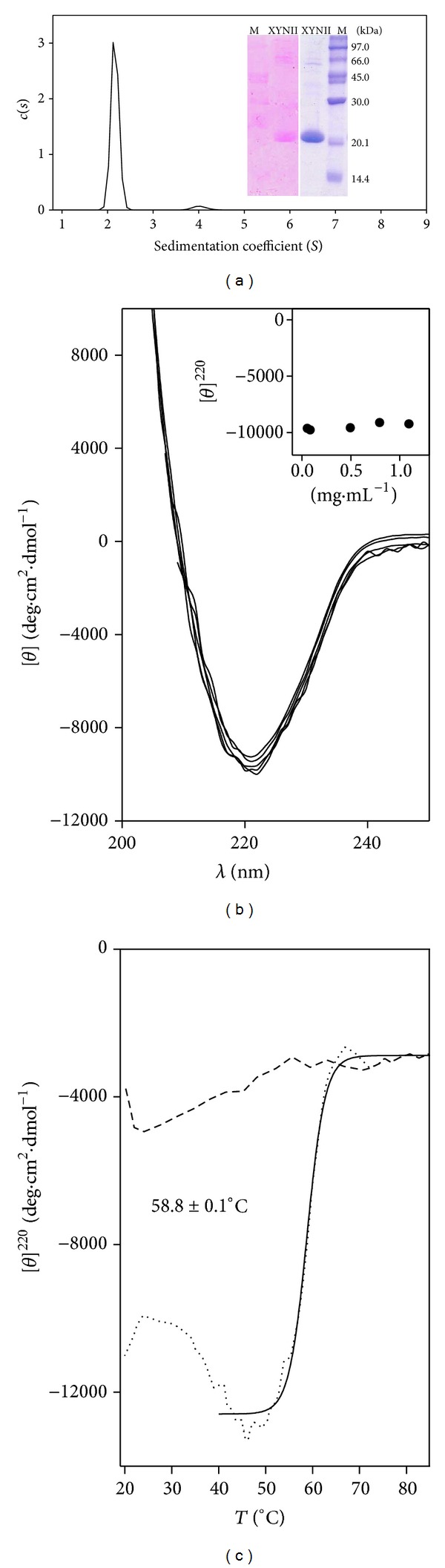
Hydrodynamic, electrophoretic, and structural characterization of xylanase. (a) Sedimentation coefficient distribution of xylanase. Inset: SDS-PAGE gel followed by glycoproteins or Coomassie staining of xylanase (XYN II) and molecular markers (M). (b) Far-UV circular dichroism spectrum of 0.06–1.1 mg*·*mL^−1^ xylanase. Inset: dependence of the molar ellipticity at 220 nm on protein concentration. (c) Plot of [*θ*]^220^ with increasing temperature (dotted line). The solid line is a fit of data to (1) and the dashed line was obtained by cooling the unfolded protein after the T ramp.

Sedimentation velocity analysis of xylanase II from* Trichoderma reesei* was carried out to get insight into the oligomeric state of the protein. The sedimentation velocity distribution *c*(*s*) of the sample is plotted in Figure 1(a). A main species accounts for 94.5% of the detected protein preparation, with the raw sedimentation coefficient being *s* = 2.162 S, the sedimentation coefficient at 20°C in water (standard conditions) being *s*_20,w_ = 2.499 S, and the molecular mass being *M* ~ 21.6 kDa with the best frictional ratio *f*/*f*0 = 1.12 and a Stokes radius of 2.82 nm. This species is compatible with xylanase monomer. The remaining protein is seen as a residual peak with *s* = 4.02 S, *s*_20,w_ = 4.65 S, and *M* ~ 55 kDa. The glycosylation state of xylanase was checked by carbohydrate staining of the gel after SDS-PAGE. Results in Figure 1(a) (inset, left panel) show that the enzyme is glycosylated. After Coomassie staining of the gel, the apparent molecular mass of the enzyme is 23.8 kDa (Figure 1(a), inset, right panel) which is higher than the theoretical one, 20,842 Da, since glycosylation affects the electrophoretic mobility of proteins.

### 3.2. Secondary Structure of Xylanase

The secondary structure of xylanase was studied by CD spectroscopy.  Figure 1(b) depicts the CD spectra of the enzyme recorded at 0.06–1.1 mg*·*mL^−1^ protein and 20°C. The spectra superimpose indicating the lack of dependence of the molar ellipticity at 220 nm ([*θ*]^220^) on protein concentration (Figure 1(b), inset), confirming the monomeric character of the enzyme. The CD profile is typical of a *β*-rich protein with a minimum at ~218–220 nm. The calculated secondary structure content was 40.9 ± 3.5% *β*-sheet, 19.6 ± 0.8% α-helix, 19.5 ± 1.2% turn, and 19.5 ± 3.4% random. The high content in *β*-sheet structure agrees with published data for XYN II from* T. reesei* containing three antiparallel *β*-sheets and one α-helix with the active site lying between the second and the third sheets [34, 35].

CD spectroscopy has been employed to investigate structural changes during the thermal unfolding of xylanase. The ellipticity at 220 nm was recorded as the temperature was increased and the [*θ*]^220^ versus temperature is depicted in Figure 1(c) (dotted line). The [*θ*]^220^ decreases with temperature to reach a minimum around 48–50°C, indicative of an increase in structural stability and coincident with the optimum temperature for catalysis [28]. An ulterior increase in temperature is followed by a sharp rise of [*θ*]^220^ in an apparent cooperative transition (the enzyme unfolding). The fitting of the data from 40°C to 85°C to (1) is the solid line. The midpoint transition is 58.8 ± 0.1°C. After the T ramp, xylanase was cooled and the data from the cooling temperature ramp (Figure 1(d), dashed line) indicate that the enzyme is unfolded and has not recovered its secondary structure upon cooling. As the thermal unfolding of xylanase is an irreversible process, the thermodynamic analysis of the data is not feasible, and thus the midpoint transition is only an apparent melting temperature (*T*_*m*_).

### 3.3. Tertiary Structure of Xylanase

**Figure 2:**
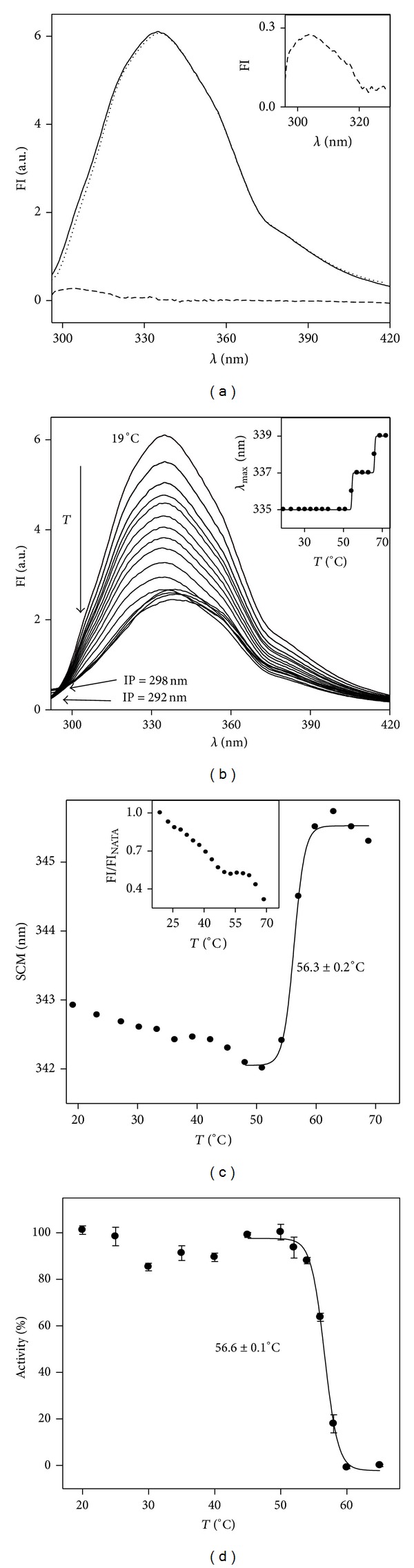
Fluorescence emission and catalytic behaviour of xylanase. (a) Fluorescence intensity emission spectrum of 0.09 mg*·*mL^−1^ xylanase in arbitrary units (a.u.) recorded at 20°C upon excitation at 275 nm (solid line), normalized trp residues emission (dotted line), and tyr residues emission (dashed line). Inset: detail of tyr residues emission at low wavelength. (b) Emission spectra of 0.09 mg*·*mL^−1^ xylanase recorded from 19°C to 70°C upon excitation at 290 nm. IP means isofluorescent point. Inset: dependence of maximum emission wavelength on temperature. (c) Variation of the xylanase SCM with temperature. The line is a fit of data above 48°C to (1). Inset: normalized xylanase to NATA fluorescence emission ratio versus temperature. (d) Percent residual activity measured in standard conditions (55°C) of 1.3 *μ*g xylanase after being preincubated for 30 min at the indicated temperatures. Data obtained at 45–65°C were fitted (solid line) to (1).

The tertiary structure of the enzyme was studied by fluorescence spectroscopy. The fluorescence emission spectrum after excitation at 275 nm is shown in Figure 2(a) (solid line) as well as the normalized emission spectrum of tryptophan residues (excitation at 290 nm, dotted line) and the emission spectrum of tyrosine residues (dashed line). The global spectrum is dominated by the trp emission contribution and the maximum emission occurs at 335 nm which indicates that the protein is folded with its trp residues buried in a hydrophobic environment. There is a very small contribution of the tyr residues to the global emission spectrum (see inset for details). This contribution, lower than expected for a trp : tyr = 6 : 17, is also typical of a properly folded protein and is probably due to either the existence of fluorescence resonance energy transfer (FRET) from tyr to nearby trp residues or quenching of tyr fluorescence by other close-side chains.

The effect of increasing the temperature on xylanase tertiary structure was checked by fluorescence spectroscopy. The fluorescence emission spectra of xylanase were recorded as the temperature of the sample increased up to 70°C and results are depicted in Figure 2(b). The fluorescence intensity decreases as the temperature is raised, whereas the maximal wavelength at which emission occurs is displaced from 335 nm at 19°C to 339 nm at 70°C (Figure 2(b), inset) indicating that the tryp residues of the enzyme have moved to a more polar environment. There are two isofluorescent points (IP) as the spectra do not cross each other in one point, but in two points (292 nm and 298 nm). This indicates that the unfolding process regarding the trp environment is not a two-state transition (only one IP would be observed). The plot of *λ*_max⁡_ versus temperature (Figure 2(b), inset) shows two thermal transitions in the 19–60°C and 57–72°C intervals (Table 1), confirming the non-two-state thermal unfolding of xylanase. Data of both temperature intervals were fitted to (1) with *λ*_max⁡_ as *Y*, but the correlation coefficients (*r*) were 1, which indicates shortage of data for fitting; that is, the experimental number of points was the minimum points that could be fitted and thus the fitting was perfect. Then, we consider midpoint transitions 54.3°C and 66°C, respectively, for both temperature intervals, as they are the only values existing between two plateaux.

On the contrary, when the spectral centre of mass (SCM, an intensity-weighted average emission wavelength) calculated with (2), was depicted versus temperature, only one transition was observed above 50°C (Figure 2(c)). The fitting of the data to (1) (solid line, *Y* is SCM) indicates apparent cooperativity in the unfolding with a midpoint transition of 56.3 ± 0.2°C (Table 1), quite close to the midpoint transition obtained in the CD transition in Figure 1(c). Around 50°C, the SCM shows a minimum value, which is coincident with the minimum value of [*θ*]^220^ observed in Figure 1(c), pointing to maximal protein stabilization around 50°C regarding both tertiary and secondary structures. To check the effect of temperature on the fluorescence intensity emission of trp residues, the fluorescence emission of N-acetyl tryptophanamide (NATA) at the same concentration than the concentration of trp residues obtained in the protein (25.92 *μ*M) was recorded upon excitation at 290 nm [33]. The ratio FI/FI_NATA_ at 339 nm was 1.28 which is higher than the theoretical value, 1, pointing again to the energy transfer from tyr to trp residues. The ratio FI/FI_NATA_ was normalized to 1 and the effect of the rising temperature on the normalized ratio is depicted in Figure 2(c) (inset). The shape of the curve shows a decrease of the intensities ratio with temperature followed by plateau around 55°C, which confirms that, regarding the trp environment, the unfolding of xylanase is not a two-state process.

### 3.4. Xylanase Stability to Temperature

Figure 2(d) depicts the xylanase residual enzymatic activity at 55°C after being preincubated at 25–70°C. Above 50–55°C, the loss of residual activity with increasing temperature is described by an apparent cooperative transition. Data were fitted to (1), with *Y* being the percent residual activity after being preincubated at each temperature and *Y*_*N*_ being the activity of the nonpreincubated protein. The midpoint transition of the curve, 56.6 ± 0.1°C (Table 1), agrees with the midpoint transition obtained by fluorescence (56.3 ± 0.2°C, Figure 2(c)) and with the published value (56.2 ± 0.4°C) [36] and is close to the midpoint transition obtained by CD (58.8 ± 1°C, Figure 1(c)).

### 3.5. Effect of Additives on Structure and Activity of Xylanase

The effect of three additives, sorbitol, soluble xylan, and d-glucono-1,5-lactone (the last two are enzyme ligands), on xylanase structure and activity has been studied. Sorbitol, a polyol of 6 carbons, has been employed previously to protect xylanase from thermal inactivation, finding that the enzyme increased its half-life 112 times in the presence of 2 M sorbitol at 60°C [28]. Insoluble xylan, the substrate of xylanase, is not suitable for structural or calorimetric studies, and therefore soluble xylan was employed. Soluble xylan is a heterogeneous mixture of xylooligosaccharides of several sizes and its composition varies according to the xylan source and the method employed to solubilize it [37, 38]. The xylooligosaccharides can act on xylanase either as ligands, as substrates (medium and short saccharides), or as inhibitors (very short saccharides), just as the reaction products do. d-Glucono-1,5-lactone (GL), a powerful inhibitor of glycosidases, is a noncompetitive inhibitor of xylanase (see Supplementary Material available online at http://dx.doi.org/10.1155/2014/708676). GL was used when mutarotation was finished (about one hour after being solved) in order to always employ the same mixture of lactone and d-gluconic acid forms [30].

#### 3.5.1. Effect of Additives on Xylanase Secondary Structure

**Figure 3:**
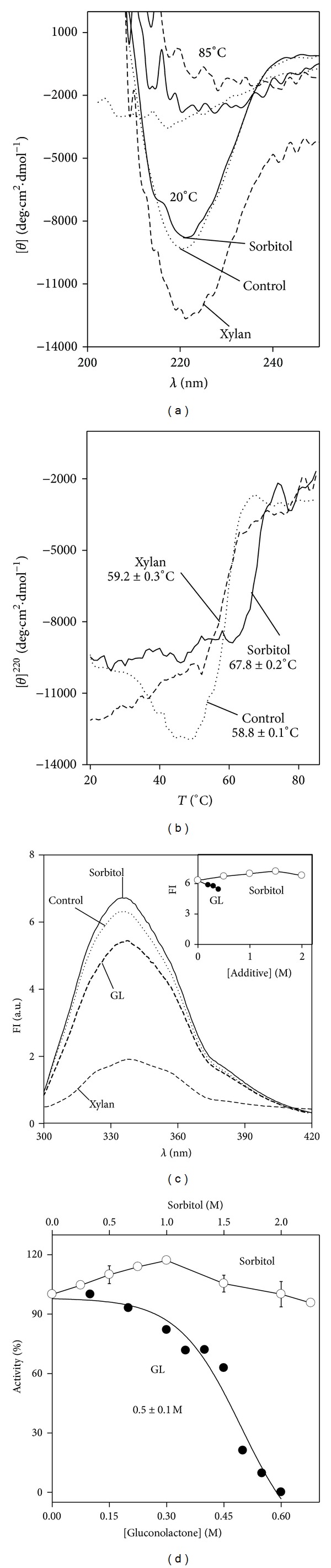
Effect of additives on the structure and activity of xylanase. (a) Far-UV CD spectra of 0.09 mg*·*mL^−1^ protein (solid line), with 2 M sorbitol (dotted line) and with 0.5% (w/v) soluble xylan (short dashed line) at 20°C. After the T ramp, the spectra of samples with or without additives were recorded at 85°C. (b) Plot of [*θ*]^220^ with increasing temperature of xylanase (dotted line), in the presence of 2 M sorbitol (solid line) and 0.5% (w/v) soluble xylan (short dashed line). Data were fitted to (1). (c) Fluorescence emission spectra of 0.09 mg*·*mL^−1^ xylanase (dotted line), with 2 M sorbitol (solid line), 0.4 M GL (long dashed line), or 0.5% soluble xylan (short dashed line). Inset: dependence of the fluorescence intensity on sorbitol (void circles) and GL (filled circles) concentration. (d) Effect of sorbitol (void circles) and GL (filled circles) on the activity of xylanase. GL data were fitted to (1).

The effect of 2 M sorbitol on xylanase secondary structure has been checked and results are depicted in Figure 3(a) at 20°C (solid lines). The intensity of the CD signal varies very little with respect to control spectrum (dotted line). The *β*-sheet content (50.80 ± 2%) with sorbitol was increased, and the α-helix (8.70 ± 2.1%) was decreased regarding control values (40.9 ± 3.5 and 19.6 ± 0.8%, resp.), while turn and random contents did not vary. The effect of temperature on the [*θ*]^220^ of xylanase with 2 M sorbitol is depicted in Figure 3(b) (solid line). The lack of the deep hollow that the control shows at ~50°C (dotted line) indicates that sorbitol hinders the effect that the increasing temperature has on the secondary structure of xylanase around 50°C. In addition, the temperature at which the apparent cooperative transition with sorbitol begins is 60°C, ~10°C higher than the temperature obtained without sorbitol (control, 50°C). The fitting of the data with sorbitol to (1) gave a transition point of 67.8 ± 0.2°C, more than 8°C higher than in the absence of sorbitol. This suggests that the stabilization that the secondary structure of xylanase suffers in the presence of sorbitol above 50°C may justify the role of sorbitol as thermoprotector. After the T ramp, the spectra were recorded at 85°C with sorbitol (Figure 3(a), solid line) and without it (Figure 3(a), dotted line), both showing the typical features of an irreversible unfolded protein since the spectra did not change after recording them upon cooling to 20°C (not shown). Figure 3(a) depicts the CD spectrum of xylanase at 20°C in the presence of 0.5% (w/v) soluble xylan (short dashed line), showing a notable decrease in the intensity of the CD signal regarding control (dotted line) around 220 nm, and also at higher wavelengths, pointing either to stabilization of the secondary structure or to a decreased contribution of aromatic residues to the CD signal, due to quenching. The secondary structure content of xylanase in the presence of soluble xylan gave values so different through the three methods employed that the calculation of the mean value was discarded. The record of the molar ellipticity at 220 nm with increasing temperature (Figure 3(b), short dashed line) shows that the apparent cooperative transition almost parallels the control one (dotted line) and the apparent *T*_*m*_ = 59.2 ± 0.3°C obtained when data were fitted to (1) was close to the apparent *T*_*m*_ obtained in the control sample. The effect of 0.4 M GL on the secondary structure of xylanase could not be determined, due to the noise in the CD signal at low wavelengths.

#### 3.5.2. Effect of Additives on Xylanase Tertiary Structure

Regarding the tertiary structure of xylanase, sorbitol (0.5 to 2 M) increased very slightly its fluorescence emission at 20°C (void circles in Figure 3(c), inset). The fluorescence emission of xylanase with soluble xylan decreased to a great extent (Figure 3(c), short dashed line) indicating that the trp emission has been strongly quenched. This result is not surprising since one of the six trp residues of xylanase (^18^trp) lies at the active-site cleft and contributes to ligand binding by being packed against the xylose ring, which probably triggers the conformational change determined by X-ray crystallography [39]. The quenching of the emission must also be the cause of the changes observed in the CD spectrum in Figure 3(a) as suggested above. The effect of GL on the tertiary structure of xylanase is depicted in Figure 3(c) (long dashed line). GL (0.2 to 0.4) decreased slightly the fluorescence emission of xylanase, with a ~15% loss at 0.4 M (filled circles in Figure 3(c), inset).

#### 3.5.3. Effect of Additives on Xylanase Activity

The residual enzymatic activity in the presence of sorbitol is shown in Figure 3(d) (void circles). At 1 M sorbitol, the activity is raised ~17% to regain control values at 2 M sorbitol. Therefore, it seems that the main thermal stabilizing effect of sorbitol (above 50°C) affects the secondary but not the tertiary structure of xylanase. Regarding the effect of GL on the catalytic activity of xylanase, results in Figure 3(d) (filled circles) show inhibition above 0.2 M and no activity was present at 0.6 M. The midpoint transition is 0.5 ± 0.1 M GL through fitting data to (1), substituting *T* and *T*_*m*_ by GL (concentration at any point) and GL_1/2_ (GL concentration at the midpoint of the curve), respectively, with *Y* being the percent residual activity.

### 3.6. Calorimetric Behaviour of Xylanase

Differential scanning calorimetry (DSC) was employed to investigate the thermodynamic characterization of the structural changes accompanying the xylanase unfolding process at high and low protein concentration.

#### 3.6.1. Irreversible Denaturation of Xylanase

**Figure 4:**
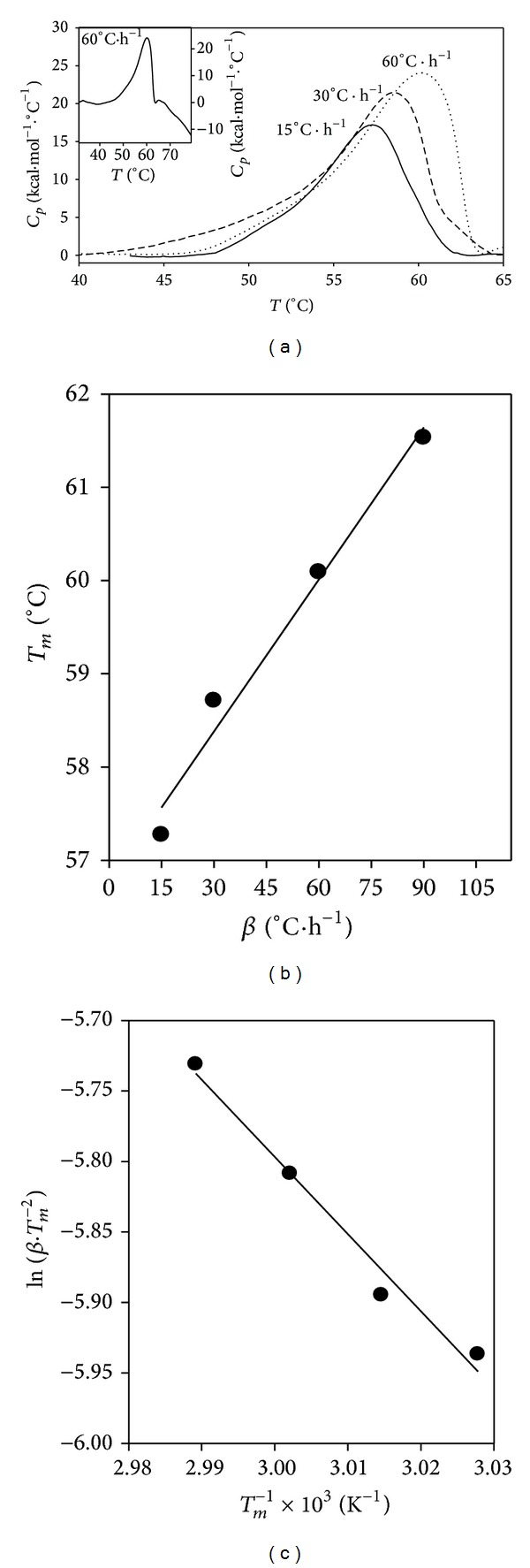
Irreversible thermal unfolding of xylanase followed by DSC. (a) Variation of the excess heat capacity (*C*_*p*_) of 1.1 mg*·*mL^−1^ xylanase with the temperature at a scan rate of 60°C*·*h^−1^ (dotted line), 30°C*·*h^−1^ (dashed line), and 15°C*·*h^−1^ (solid line) in the 40–65° interval. Inset: detail of the thermogram at 60°C*·*h^−1^ in the 31–79°C interval. (b) Variation of the scan rate (*β*) with the apparent *T*_*m*_. The line is a fit by linear regression. (c) Determination of the activation energy through the scan rate dependence on *T*_*m*_ (solid line) by fitting data to (3).

The variation of the protein excess heat capacity (*C*_*p*_) with the temperature increase at 1.1 mg*·*mL^−1^ protein in the 30–80°C interval is shown in Figure 4(a). The process is characterized by a single cooperative endothermic peak centred at a transition temperature (the maximum excess heat capacity at which half of the enzyme is unfolded) of 60.1°C at a scan rate of 60°C*·*h^−1^ (dotted line). The thermal denaturation of xylanase is irreversible as no endothermic peak was observed by rescanning the sample cooled after the first run (not shown). The protein unfolds irreversibly due to aggregation since an exothermic contribution is seen at temperatures higher than 65°C (Figure 4(a), inset). The dependence of the transition temperature (apparent *T*_*m*_) on the scan rate for 1.1 mg*·*mL^−1^ xylanase was also studied in the 37–80°C interval at several other scan rates. The resulting thermograms are shown in Figure 4(a) where we see that the greater the scan rate (*β*), the higher the transition temperature. The dependence of the apparent *T*_*m*_ on the scan rate is linear (Figure 4(b)) indicating that the thermal unfolding of xylanase is irreversible and follows first order kinetics. Under these conditions, the unfolding reaction is under kinetic control and no thermodynamic information can be extracted from the DSC experiments as the reaction is not in thermodynamic equilibrium [40]. This result agrees with published results for xylanase II from* T. reesei* [18]. The apparent *T*_*m*_ and the calorific enthalpy change, Δ*H*_cal_ (the area under the endothermic peak), values are summarized in Table 2. As the final temperature in the calorimetric profile was 80°C and it is known that high temperatures may hinder reversibility by promoting aggregation processes, we looked for a possible reversible denaturation by initiating the second scan just after the drop of the endothermic peak (~63°C at 60°C*·*h^−1^). However, a flat profile was obtained again for the second scan (not shown). This means that the DSC transition is strongly rate-limited [40].

Irreversible unfolding behaviour has been found for many globular proteins and in several cases equilibrium thermodynamics has been applied to the analysis of calorimetric curves, although the kinetic control of the process did not allow this treatment. Therefore, when an irreversible process occurs, the kind of information that can be obtained from calorimetric experiments depends on each particular situation [41]. According to this, we have analyzed, in the irreversible denaturation of xylanase, the effect of the scan rate on the apparent *T*_*m*_. The purpose was to determine the apparent activation energy (apEa) of the irreversible step by fitting data (Figure 4(c)) to (3). The apparent activation energy for the irreversible unfolding of xylanase, apEa = 45.51 kJ*·*mol^−1^, is quite a low value and indicates that the enzyme shows a low energetic barrier for its thermal inactivation. This value cannot be compared with the inactivation energies obtained above 50°C in the Arrhenius plot (supplementary data) since the substrate (always a stabilizer) was present, in contrast with the lack of substrate in the actual calorimetric measures. Regarding the pertinence of applying (3) to the calorimetric data obtained for xylanase thermal unfolding, as we did, we must remember that this equation was initially proposed for irreversible two-state processes [40] and later intended to be the most unequivocal test for detecting the two-state irreversible model [42]. However, calorimetric data of xylanase from* Bacillus circulans* showed the linear dependence described in (3) but did not fit to an irreversible two-state model [17] making the abovementioned criterion not necessarily of a general validity. Therefore, we employed (3) to fit the calorimetric data of the xylanase irreversible processes, disregarding whether the protein unfolds through a two-state or through a non-two-state process.

#### 3.6.2. Reversible Denaturation of Xylanase

**Figure 5:**
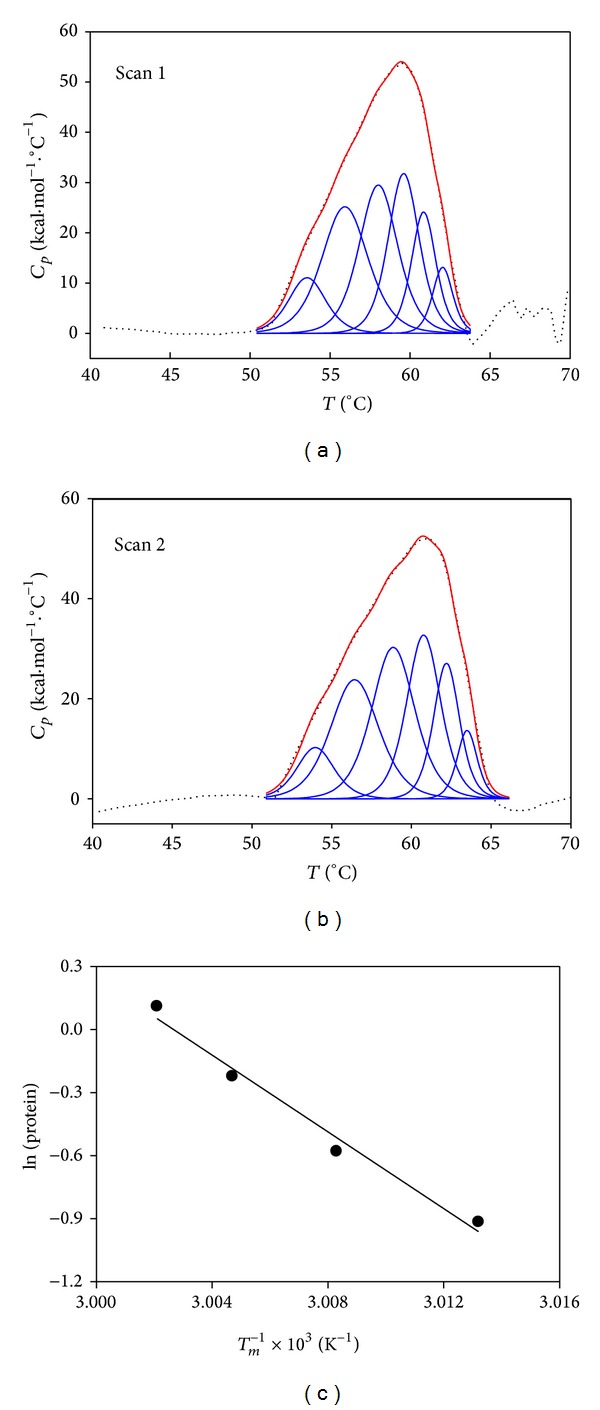
Reversible thermal unfolding of xylanase followed by DSC. (a) Variation of the excess heat capacity of 0.56 mg*·*mL^−1^ xylanase with temperature at 60°C*·*h^−1^ scan rate. The data (black dotted line) were fitted to the non-two-state model obtaining the cumulative Gauss curve through the data (red solid line). Six blue solid lines were obtained by deconvolution analysis with (4). (b) Same as in (a) except that the data correspond to the second scan of the sample. (c) Dependence of *T*_*m*_ on xylanase concentration, 0.4 and 0.56 mg*·*mL^−1^ (reversible process), and of apparent *T*_*m*_ on 0.8 and 1 mg*·*mL^−1^ (irreversible process). The line is an itered fit of the data to (5).

Thermal denaturation involves the kinetics of unfolding and aggregation, and thus the transitions depend not only on the heating rate but also on protein concentration. As xylanase unfolding at 1.1 mg*·*mL^−1^ was irreversible whatever the scan rate tested and since aggregation is a concentration-dependent process, we decreased the protein concentration in the calorimetric cell, intending to reduce the rates of aggregation of the unfolded xylanase by reducing the frequency of intermolecular contacts responsible for triggering protein aggregation. To this, DSC experiments were carried out at 0.8, 0.56, and 0.4 mg*·*mL^−1^ protein but maintaining a high scan rate (60°C*·*h^−1^) which is usually considered the most appropriate for seeking reversibility [43]. At 0.8 mg*·*mL^−1^ protein, the unfolding process was still irreversible (not shown) but, at 0.56 and 0.4 mg*·*mL^−1^ protein, the second and the third scans also showed endothermic peaks indicating that the protein was folded, once cooled after the first run, and thus it was able to unfold again as the temperature was increased.  Figure 5 shows the first (Figure 5(a)) and second (Figure 5(b)) scans of 0.56 mg*·*mL^−1^ xylanase subjected to DSC. The area under the peak (Δ*H*_cal_) is higher in the first scan (481.9 kCal*·*mol^−1^, Table 3) than in the second scan (405.7 kCal*·*mol^−1^, not shown) indicating that ~16% of the protein was unable to fold after the first scan.

As the *C*_*p*_ curve reflects the energetic variations associated with differently populated states in thermodynamic equilibrium with each other, the number of intermediate states and the thermodynamic variables associated with the transitions can be calculated by using an appropriate mechanical-statistical deconvolution algorithm [44]. The reversible transitions were subjected to thermodynamic deconvolution analysis and the thermogram of each successive scan was fitted to the non-two-state model with six peaks according to (4). Six regions or subdomains (1 to 6) should unfold independently in the protein as reversible two-state processes indicating that some regions of the molecule may be thermodynamically more stable than others. Each subdomain will have a term in (4). As illustrated in Figure 5, the multiple Gauss model accounts well for the thermograms of xylanase since the cumulative Gauss curve (red solid line) describes the experimental DSC curve (black dotted line) on the first scan (Figure 5(a)) and second scan (Figure 5(b)) satisfactorily. The six peaks are depicted as blue solid lines.

The values obtained for *T*_*m*_, Δ*H*_cal_, and Δ*H*_VH_ (van't Hoff enthalpy change) for each individual transition in the first scan are summarized in Table 3. Δ*H*_VH_ depends on the shape of the peak and assumes a true two-state reversible equilibrium. For this reason, it was not determined for the global endotherm which fits to the non-two-state model (ND). Δ*H*_VH_ is the enthalpy change per mole of cooperative unit [43] and is independent of the amount of protein in solution, so that it is considered a measure of the cooperativity of the transition: the greater the cooperativity, the sharper the transition and the greater the Δ*H*_VH_. Considering the parameters values in Table 3, the ratio Δ*H*_VH_/Δ*H*_cal_ is far higher than 1 for each transition (~1 should be obtained in a two-state process). This can indicate either that the unfolding process does not correspond to all native tertiary structure contacts or that intermolecular interactions between native or denatured states are taking place, or both, as suggested for other proteins [45]. Table 1 summarizes the apparent *T*_*m*_ of xylanase obtained by CD, fluorescence emission, residual activity, and DSC (irreversible calorimetric transition, 1.1 mg*·*mL^−1^ protein, 30°C*·*h^−1^) and the *T*_*m*_ obtained in the reversible process (0.56 mg*·*mL^−1^ protein, same scan rate). The values of the transition temperatures in DSC studies at high and low protein concentration, 58.71°C and 58.9°C, respectively, are close to the value obtained in CD studies, whereas the transition temperature in fluorescence experiments approaches the temperature obtained in the residual-activity study. This indicates that the calorimetric transition of the enzyme is bound to the loss of secondary structure, whereas the decay of the activity is related to the loss of the tertiary structure.

#### 3.6.3. Are Dimerization and Glycosylation Also Responsible for the Multiplicity of Transitions in the Xylanase Reversible Unfolding?

Alternatively, if the protein molecules unfold, for instance, as dimers, then the transition will be sharper and Δ*H*_VH_ will be greater than expected if only the monomer was considered for normalizing the protein concentration in the Δ*C*_*p*_ calculus [43] and Δ*H*_VH_/Δ*H*_cal_ will be increased. Xylanase II has been found partly dimerized at 50°C [13], and thus its thermal unfolding may be more complex than we presume if only monomers are present in the solution. A well-known test to determine the molecularity of the reaction, that is, whether only monomers are taking part in the unfolding process or whether oligomers are also present, is to check if the *T*_*m*_ changes as the protein concentration increases. If this is the case, the calorimetric data obtained at several protein concentrations must be fit to (5) [40, 41]. Results depicted in Figure 5(c) show a linear dependence of ln[protein] on *T*_*m*_ (0.4 and 0.56 mg*·*mL^−1^, reversible process) and on apparent *T*_*m*_ (0.8 and 1.1 mg*·*mL^−1^, irreversible process). The molecularity value (*μ*) obtained from the slope is 1.05, indicating that practically one xylanase molecule is involved in the unfolding process; that is, the transition is mainly monomolecular. However, as there is a small dependence of *T*_*m*_ on protein concentration, it could indicate that a part of protein molecules dissociate as dimers. About 10–30% of xylanase II molecules form dimers at 50°C [13], and this complicates the interpretation of results since we could have committed several errors while processing the data. First, we have considered only monomers to normalize protein concentration for processing calorimetric data but, at ~50°C, there would also be dimers in solution. Second, monomers would unfold above 50°C but dimers must dissociate before unfolding, and thus the fitting may accord to a different equation which accounts for oligomer dissociation. As the sedimentation velocity of xylanase could not be determined at 50°C, thus we heated the sample at 50°C, and then we proceeded to sedimentation analysis at 20°C. No other peaks than those depicted in Figure 1(a) were found (results not shown). This indicates that dimers formed at 50°C, if any, would dissociate when the temperature is decreased.

In addition, we must consider the effect of glycosylation on xylanase thermal stability. Protein stability is, in general, increased with glycosylation although nowadays it is controversial whether the stabilizing effect of sugars is enthalpic in origin (glycan establishing new interactions with amino acids) or entropic in origin (glycans with chaperone-like activity). It seems that the degree of stabilization depends on the position of the glycan but only very weakly on the size of the glycan [46]. Sometimes, as occurred to xylanase from* Thermpolyspora flexuosa,* glycosylation of an asn residue exposed in a loop decreases its thermal stability [47]. Xylanase II from* T. reesei* holds two to three potential glycosylation sites determined by homology [13, 48]. On the other hand, xylanase II from the strain Rut C 30 contains less than 1% of bound carbohydrate [22] although the enzyme was also found not glycosylated [49]. Other results showed that about 60% of xylanase II was not glycosylated with *M* = 20,825 Da and about 30% was glycosylated with N-acetyl glucosamine (GlcNAc) [13]. Also, a nonglycosylated form of the enzyme expressed on yeast showed a molecular mass of 21 kDa [50]. In view of the data commented above, one or several peaks in Figure 5(a), which we attributed initially to the unfolding of xylanase domains or subdomains, could be due to the unfolding of glycosylated forms of the enzyme. If we assume that glycosylation stabilizes proteins, those peaks could be the ones with the highest *T*_*m*_ (Table 3).

### 3.7. Effect of Ligands on Xylanase Calorimetric Transition

The application of Le Chatelier's principle shows that if a ligand (usually a small molecule) binds preferentially to the folded or native form of a protein, then the folded state will be stabilized and the unfolding of the protein will be less favourable as the ligand concentration is increased. Conversely, ligands that bind preferentially to the unfolded protein stabilize the unfolding [43]. We have studied the effect of ligands on xylanase thermal unfolding employing a substrate (soluble xylan) and an inhibitor (GL) with a protein concentration (1 mg*·*mL^−1^) at which irreversible thermal unfolding was observed in absence of ligands.

#### 3.7.1. Effect of d-Glucono-1,5-Lactone

**Figure 6:**
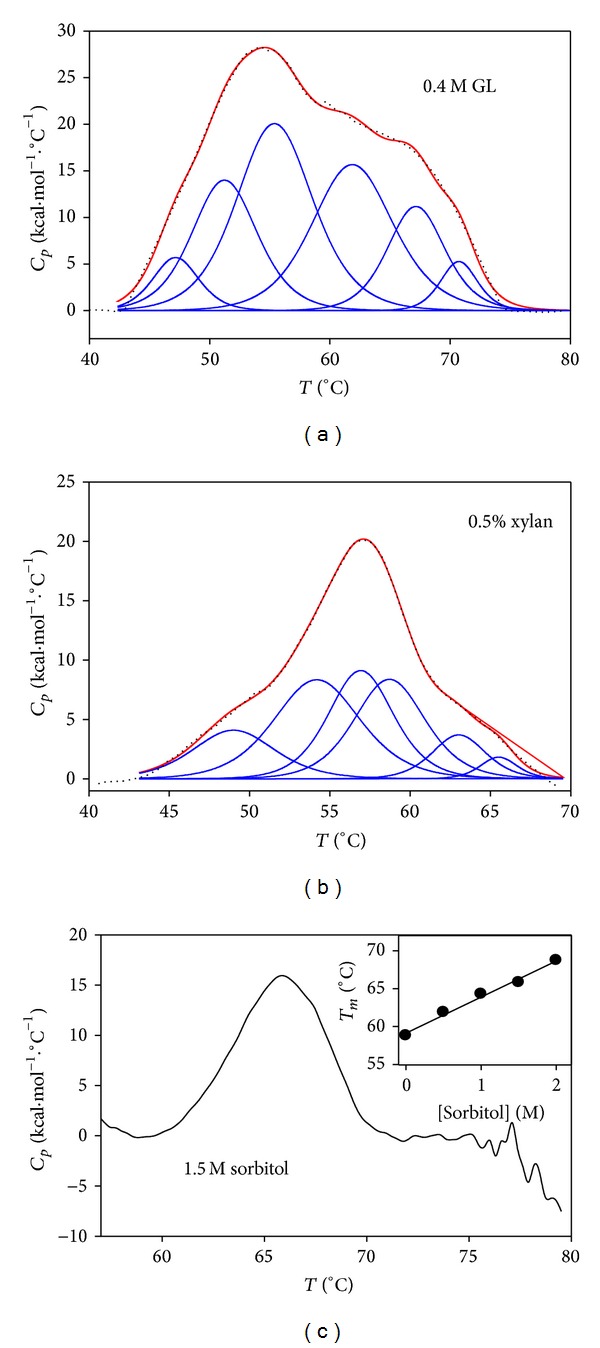
Effect of additives on the thermal unfolding of xylanase followed by DSC. (a) The thermogram of xylanase (1 mg*·*mL^−1^) was obtained at 15°C*·*h^−1^ scan rate in the presence of 0.4 M GL. (b) The thermogram of 1 mg*·*mL^−1^ protein was obtained in the presence of 0.5% (w/v) soluble beechwood xylan at 15°C*·*h^−1^ scan rate. Dotted and coloured solid lines in (a) and (b) mean the same as in Figure 5(a). (c) Irreversible calorimetric transition of 0.4 mg*·*mL^−1^ xylanase containing 1.5 M sorbitol at 60°C*·*h^−1^ scan rate. Inset: linear dependence of the apparent *T*_*m*_ on sorbitol concentration (0.5–2 M).

As GL inhibits xylanase catalytic activity above 0.2 M (Figure 3(d)), DSC experiments were carried out with 0.4 M GL in the calorimetric cell. Other conditions were 1 mg*·*mL^−1^ protein and a scan rate of 15°C*·*h^−1^, under which an irreversible transition was observed in control samples, in the absence of GL. The resulting thermogram, in the presence of GL, is depicted in Figure 6(a). The thermal unfolding process of xylanase has become reversible as the second scan was not flat (not shown) and no exothermic contribution was detected at higher temperatures, which indicates that GL hinders protein aggregation. The *T*_*m*_ of the global calorimetric process is 54.1°C (Table 4), which is a transition temperature lower than that obtained without GL and with the same scan rate (57.27°C, Table 2). An explanation of this low value must be the particular shape of the thermogram, which features a broad shoulder at higher temperatures, indicating that overlapping thermal processes taking place are beginning to segregate. As the process with GL was reversible, the endothermic peak of the thermogram was fitted to the non-two-state model, and the deconvolution analysis of the data with (4) gave six peaks whose *T*_*m*_ values are summarized in Table 4. If the assumption that the peaks with the highest *T*_*m*_ (transitions 5 and 6) are due to glycosylated enzyme forms is correct, then GL would stabilize preferentially glycosylated xylanase forms.

#### 3.7.2. Effect of Soluble Xylan

Substrates are known to be stabilizers of the enzymes as shows xylanase from* T. flexuosa* [47]. The stabilizing effect of the substrate can arise from the additional free energy required to remove the ligand prior to unfolding, together with an additional contribution (*RT* · ln⁡[Ligand]) from the entropy of adding the ligand to the bulk solvent [43]. The calorimetric unfolding of xylanase was carried out in the presence of 0.5% (w/v) soluble xylan and 1 mg*·*mL^−1^ protein at 15°C*·*h^−1^ scan rate. Results of the first scan are shown in Figure 6(b) where we see that there is no exothermic contribution in the thermogram, indicating that xylan hinders protein aggregation. As the second scan is not flat (not shown), we conclude that the thermal unfolding of xylanase with xylan has become reversible under protein concentration and scan rate conditions that, in absence of xylan, promote its irreversible thermal transition. The *T*_*m*_ of the reversible process in the presence of xylan (56.9°C, Table 4) is very close to the apparent *T*_*m*_ of the irreversible process in its absence (57.27°C, Table 2), instead of being higher as expected for a ligand which is expected to bind to the folded form of the enzyme. Data from the endothermic peak in the thermogram of Figure 6(b) were fitted to (4) and to the non-two-state model and the deconvolution analysis gave 6 peaks whose transition temperatures are summarized in Table 4. It may be that to account for this behaviour we must consider, in addition to the effect of temperature on the enzyme itself, several other processes taking place in the calorimetric cell. First, medium and short xylooligosaccharides of soluble xylan would act as substrate being converted into products at a rate increasing with the increase in temperature (see the Arrhenius plot in supplementary data), and thus its binding to the folded enzyme will stabilize it. Second, the reaction products of the enzymatic reaction taking place at the calorimetric cell can also bind to the enzyme as inhibitors. In the third place, the very short xylooligosaccharides that soluble xylan contains may also bind to the enzyme as inhibitors, since they are also the reaction products. The chemical mechanism of xylanase involves the formation of an intermediate glycosyl-enzyme as glycosidases do [51], but, to our knowledge, there are no available data regarding its kinetic mechanism. It may be that the enzyme follows an Ordered Uni Bi mechanism as *β*-xylosidase from* T. reesei* does [52]. If this is the case, the first sugar leaving the active centre of the enzyme would act as mixed dead-end and product inhibitor (it would bind to the xylosyl-enzyme) and the second sugar leaving the active centre would be a competitive inhibitor (it would bind to the free enzyme). This would create a complex situation in the calorimetric cell since the reaction products, usually at lower concentration than the substrate, in this case are at high concentration since they are incorporated into the cell with soluble xylan. Moreover, the binding of those sugars to unfolded enzyme forms cannot be totally ruled out.

### 3.8. Effect of Sorbitol on Xylanase Calorimetric Transition

The ability of polyols to protect enzymes against thermoinactivation has been related to their ability to decrease the water activity by reducing the “free” water molecules through water-polyol hydrogen bonding interactions, thus immobilizing the water medium [53]. To study the effect of 0.5–2 M sorbitol on the thermal denaturation xylanase by DSC, a protein concentration showing thermal reversibility in the absence of additives was employed (0.4 mg*·*mL^−1^). The thermogram of xylanase with 1.5 M sorbitol is shown in Figure 6(c), with the second scan being flat (not shown). It indicates that the reversibility shown by the thermal unfolding of xylanase at low protein concentration was lost in the presence of sorbitol. The exothermic contribution observed above 77°C in the thermogram can be attributed to protein aggregation. The irreversibility was shown at each sorbitol concentration tested, and the apparent *T*_*m*_ of the process increased linearly with sorbitol concentration (Figure 6(c), inset). The apparent *T*_*m*_ was about 10°C higher with 2 M sorbitol than in its absence (see inset in Figure 6(c)). This linear dependence agrees with previous data published for other enzymes such as lysozyme [54]. The thermal stabilization of xylanase by sorbitol that we have reported previously [28] may be due to a preferential solvent interaction effect of sorbitol which strengthens the hydrophobic interactions on xylanase, as suggested for other proteins [55]. Nevertheless, once the protein is unfolded, sorbitol may strengthen the interactions between hydrophobic pockets hidden in the native protein, propitiating protein aggregation and hindering the reversibility of the process. That is, sorbitol would stabilize xylanase against thermal unfolding but once the enzyme is unfolded, sorbitol would play against the reversibility of the process by promoting hydrophobic interactions between unfolded molecules that would lead to protein aggregation.

## 4. Conclusions

1. Combined data from far-UV circular dichroism, intrinsic fluorescence emission spectroscopy, and catalytic stability point to the thermal unfolding of xylanase II from* T. reesei* as a non-two-state process. The enzyme loses its secondary structure in an apparent cooperative process with an apparent *T*_*m*_ = 58.8 ± 0.1°C, whereas the tertiary structure and the catalytic stability show apparent *T*_*m*_ = 56.3 ± 0.2°C and 56.6 ± 0.1°C, respectively, indicating that the loss of its catalytic activity is linked to the loss of its tertiary structure and that both of them occur at lower temperature than the loss of secondary structure.
2. The calorimetric transition of xylanase studied by DSC is reversible or irreversible depending on protein concentration. Above 0.56 mg*·*mL^−1^, the process is irreversible and the apparent *T*_*m*_ shows linear dependence on the scan rate, indicating that the thermal unfolding of xylanase is under kinetic control. At lower protein concentration, the process is reversible and the experimental data were fitted to the non-two-state model and deconvoluted into six thermal transitions. Whether those transitions correspond to the thermal unfolding of either protein domains or subdomains or to either glycosylated or nonglycosylated transient dimeric and monomeric enzyme forms or to both is still an open question, although the process seems to be mainly monomolecular. The *T*_*m*_ = 59.4°C of reversible unfolding is higher than the apparent *T*_*m*_ obtained in structural or catalytic stability studies. To avoid protein aggregation when temperatures close to the optimum temperature for catalysis (55°C) are employed, xylanase should be employed below 0.56 mg*·*mL^−1^.
3. The effect of additives on the calorimetric transition of xylanase is dependent on their nature. d-Glucono-1,5-lactone, a noncompetitive inhibitor of xylanase, changes the irreversible unfolding thermal process at high protein concentration into reversible, while the thermogram shows a low *T*_*m*_ (54.1 ± 1°C), six peaks when data were deconvoluted, and a broad shoulder at the highest temperatures. A substrate, soluble xylan, also turns into reversible the irreversible unfolding of xylanase. On the contrary, the presence of sorbitol, a polyol known to act as thermal stabilizer of proteins, makes irreversible the reversible thermal unfolding of xylanase at low protein concentration and the apparent *T*_*m*_ of the process is dependent on sorbitol concentration. The addition of 2 M sorbitol allows the increase of the working temperature of xylanase close to 60°C by stabilizing the secondary structure but temperature must be kept around this value to avoid xylanase aggregation.

## Supplementary Material

Supplementary materials deal with the non-competitive inhibition of xylanase by D-gluconolactone, the inhibition constants being determined. Also, the effect of temperature on xylanase activity is presented according to the Arrhenius plot of the data, the activation and inactivation energies for the catalysis of xylan being determined.

## Figures and Tables

**Table 1:** Data from the temperature dependence of [*θ*]^220^ (Figure 1(c)), SCM (Figure 2(c)), and residual catalytic activity (Figure 2(d)) were fitted to (1) to obtain the midpoint of irreversible transitions (apparent *T*_*m*_).

	Parameter				Calorimetric transition	
	[*θ*]^220^	*λ*_max⁡^a^_	SCM	% activity	Irreversible	Reversible
*T*_*m*_ (°C)	58.8 ± 1.0	54.3 66.0	56.3 ± 0.2	56.6 ± 0.1	58.71	58.9

^a^Data in Figure 2(b) inset were fitted to (1) in the 19–60°C and 57–72°C. Midpoint transitions were obtained from the calorimetric irreversible process of 1.1 mg*·*mL^−1^ protein (apparent *T*_*m*_, Figure 4(a)) at 30°C*·*h^−1^ scan rate and from the reversible transition of 0.56 mg*·*mL^−1^ protein (*T*_*m*_, not shown) at 30°C*·*h^−1^ scan rate.

**Table 2:** Data from the irreversible calorimetric transition of 1.1 mg*·*mL^−1^ protein were taken from Figure 4(a) except for data obtained at 90°C*·*h^−1^ scan rate (not shown in Figure 4(a)). Δ*H*_cal_ was obtained integrating the area under the endothermic peaks.

	Scan rate of irreversible transition			
	15 (°C*·*h^−1^)	30 (°C*·*h^−1^)	60 (°C*·*h^−1^)	90 (°C*·*h^−1^)
*T*_*m*_ (°C)	57.27	58.71	60.09	61.53
Δ*H*_cal_ (kCal*·*mol^−1^)	112.58	172.61	183.38	293.58

**Table 3:** Data from the excess heat capacity versus temperature curves in Figure 5(a) were fitted to (4). The changes in both the calorimetric enthalpy (Δ*H*_cal_) and the van't Hoff enthalpy (Δ*H*_VH_) were obtained through the deconvolution analysis of data (*χ* ^2^/DoF = 0.86).

	Transition						
	1	2	3	4	5	6	Global
*T*_*m*_ (°C)	53.2	55.3	57.4	59.3	60.9	62.9	**59.4**
Δ*H*_cal_ (kCal*·*mol^−1^)	33.4	79.2	111.2	121.4	120.5	18.8	**481.9**
Δ*H*_VH_ (kCal*·*mol^−1^)	313.3	250.2	241.9	255.4	257.2	365.3	**ND**

ND is not determined as the global process is not a two-state process.

**Table 4:** Data from the reversible thermal unfolding of xylanase in the presence of 0.4 M GL and 0.5% (w/v) soluble xylan from Figures 6(a) and 6(b) were fitted to (4). The protein concentration was 1 mg*·*mL^−1^ and the scan rate was 15°C*·*h^−1^.

	Transition (*T*_*m*_ in °C)						
	1	2	3	4	5	6	Global
0.4 M GL	47.2 ± 0.3	51.3 ± 0.5	55.4 ± 0.6	61.9 ± 0.3	67.2 ± 0.2	70.7 ± 0.2	**54.1 **
0.5% (w/v) xylan	49.0 ± 0.4	54.2 ± 2.6	57.0 ± 1.6	58.7 ± 2.8	63.0 ± 0.3	65.5 ± 0.4	**56.9 **

## Symbols

CD: Circular dichroism
DSC: Differential scanning calorimetry
GL: d-Glucono-1,5-lactone
NATA: N-Acetyl-L-tryptophanamide
*T*_*m*_: Melting temperature.

## References

[1] Biely P (1985). Microbial xylanolytic systems. *Trends in Biotechnology*.

[2] Torronen A, Mach RL, Messner R (1992). The two major xylanases from Trichoderma reesei: characterization of both enzymes and genes. *Biotechnology*.

[3] Viikari L, Kantelinen A, Sundquist J, Linko M (1994). Xylanases in bleaching: from an idea to the industry. *FEMS Microbiology Reviews*.

[4] Ciechańska D (2001). Progress in biomodification of cellulose pulps by cellulases and xylanases. *Fibres and Textiles in Eastern Europe*.

[5] Maat J, Roza M, Verbakel J, Stam H, Santos da Silva MJ, Bosse M, Visser J, Beldman G, Kusters van Someren MA, Voragen AJG (1992). Xylanases and their application in backery. *Xylans and Xylanases*.

[6] Turunen O, Etuaho K, Fenel F (2001). A combination of weakly stabilizing mutations with a disulfide bridge in the α-helix region of Trichoderma reesei endo-1,4-*β*-xylanase II increases the thermal stability through synergism. *Journal of Biotechnology*.

[7] Xiong H, Fenel F, Leisola M, Turunen O (2004). Engineering the thermostability of Trichoderma reesei endo-1,4-*β*- xylanase II by combination of disulphide bridges. *Extremophiles*.

[8] Sriprang R, Asano K, Gobsuk J, Tanapongpipat S, Champreda V, Eurwilaichitr L (2006). Improvement of thermostability of fungal xylanase by using site-directed mutagenesis. *Journal of Biotechnology*.

[9] Zhang S, He Y, Yu H, Dong Z (2014). Seven N-terminal residues of a thermophilic xylanase are sufficient to confer hyperthermostability on its mesophilic counterpart. *PLoS ONE*.

[10] Arase A, Yomo T, Urabe I, Hata Y, Katsube Y, Okada H (1993). Stabilization of xylanase by random mutagenesis. *The FEBS Letters*.

[11] Wakarchuk WW, Sung WL, Campbell RL, Cunningham A, Watson DC, Yaguchi M (1994). Thermostabilization of the *Bacillus circulans* xylanase by the introduction of disulfide bonds. *Protein Engineering*.

[12] Shibuya H, Kaneko S, Hayashi K (2000). Enhancement of the thermostability and hydrolytic activity of xylanase by random gene shuffling. *Biochemical Journal*.

[13] Jänis J, Rouvinen J, Leisola M, Turunen O, Vainiotalo P (2001). Thermostability of endo-1,4-*β*-xylanase II from *Trichoderma reesei* studied by electrospray ionization Fourier-transform ion cyclotron resonance MS, hydrogen/deuterium-exchange reactions and dynamic light scattering. *Biochemical Journal*.

[14] Fukumura M, Tanaka A, Sakka K, Ohmiya K (1995). Process of thermal denaturation of xylanase (XynB) from *Clostridium stercorarium* F-9. *Bioscience, Biotechnology and Biochemistry*.

[15] Ruz-arribas A, Zhadan GG, Kutyshenko VP (1998). Thermodynamic stability of two variants of xylanase (Xys1) from *Streptomyces halstedii* JM8. *European Journal of Biochemistry*.

[16] Araki R, Karita S, Tanaka A, Suzuki M, Kimura T, Sakka K (2007). Thermal unfolding and modular architecture of *Clostridium stercorarium* Xyn10B. *Bioscience, Biotechnology and Biochemistry*.

[17] Davoodi J, Wakarchuk WW, Surewicz WK, Carey PR (1998). Scan-rate dependence in protein calorimetry: the reversible transitions of *Bacillus circulans* xylanase and a disulfide-bridge mutant. *Protein Science*.

[18] Jänis J, Rouvinen J, Vainiotalo P, Turunen O, Shnyrov VL (2008). Irreversible thermal denaturation of *Trichoderma reesei* endo-1,4-*β*-xylanase II and its three disulfide mutants characterized by differential scanning calorimetry. *International Journal of Biological Macromolecules*.

[19] Murakami MT, Arni RK, Vieira DS, Degrève L, Ruller R, Ward RJ (2005). Correlation of temperature induced conformation change with optimum catalytic activity in the recombinant G/11 xylanase a from *Bacillus subtilis* strain 168 (1A1). *FEBS Letters*.

[20] Roberge M, Lewis RNAH, Shareck F (2003). Differential scanning calorimetric, circular dichroism, and Fourier transform infrared spectroscopic characterization of the thermal unfolding of xylanase A from *Streptomyces lividans*. *Proteins: Structure, Function and Genetics*.

[21] Purmonen M, Valjakka J, Takkinen K, Laitinen T, Rouvinen J (2007). Molecular dynamics studies on the thermostability of family 11 xylanases. *Protein Engineering, Design and Selection*.

[22] Tenkanen M, Puls J, Poutanen K (1992). Two major xylanases of *Trichoderma reesei*. *Enzyme and Microbial Technology*.

[23] Xu J, Takakuwa N, Nogawa M, Okada H, Morikawa Y (1998). A third xylanase from *Trichoderma reesei* PC-3-7. *Applied Microbiology and Biotechnology*.

[24] Parkkinen T, Hakulinen N, Tenkanen M, Siika-aho M, Rouvinen J (2004). Crystallization and preliminary X-ray analysis of a novel *Trichoderma reesei* xylanase IV belonging to glycoside hydrolase family 5. *Acta Crystallographica D*.

[25] Tenkanen M, Vršanská M, Siika-Aho M (2013). Xylanase XYN IV from *Trichoderma reesei* showing exo- and endo-xylanase activity. *The FEBS Journal*.

[26] Acebal C, Castillon MP, Estrada P (1986). Enhanced cellulase production from *Trichoderma reesei* QM 9414 on physically treated wheat straw. *Applied Microbiology and Biotechnology*.

[27] López G, Bañares-Hidalgo A, Estrada P (2011). Xylanase II from *Trichoderma reesei* QM 9414: conformational and catalytic stability to chaotropes, trifluoroethanol, and pH changes. *Journal of Industrial Microbiology and Biotechnology*.

[28] Cobos A, Estrada P (2003). Effect of polyhydroxylic cosolvents on the thermostability and activity of xylanase from *Trichoderma reesei* QM 9414. *Enzyme and Microbial Technology*.

[29] Miller GL (1959). Use of dinitrosalicylic acid reagent for determination of reducing sugar. *Analytical Chemistry*.

[30] Gómez M, Isorna P, Rojo M, Estrada P (2001). Chemical mechanism of *β*-xylosidase from *Trichoderma reesei* QM 9414: pH-dependence of kinetic parameters. *Biochimie*.

[31] Sreerama N, Woody RW (2000). Estimation of protein secondary structure from circular dichroism spectra: comparison of CONTIN, SELCON, and CDSSTR methods with an expanded reference set. *Analytical Biochemistry*.

[32] Eisinger J (1969). Intramolecular energy transfer in adrenocorticotropin. *Biochemistry*.

[33] Richardson JM, Lemaire SD, Jacquot J-, Makhatadze GI (2000). Difference in the mechanisms of the cold and heat induced unfolding of thioredoxin h from *Chlamydomonas reinhardtii*: spectroscopic and calorimetric studies. *Biochemistry*.

[34] Törrönen A, Harkki A, Rouvinen J (1994). Three-dimensional structure of endo-1,4-*β*-xylanase II from *Trichoderma reesei*: two conformational states in the active site. *The EMBO Journal*.

[35] Törrönen A, Rouvinen J (1995). Structural comparison of two major endo-1,4-xylanases from *Trichoderma reesei*. *Biochemistry*.

[36] Jänis J, Turunen O, Leisola M, Derrick PJ, Rouvinen J, Vainiotalo P (2004). Characterization of mutant xylanases using fourier transform ion cyclotron resonance mass spectrometry: stabilizing contributions of disulfide bridges and N-terminal extensions. *Biochemistry*.

[37] Liab K, Azadi P, Collins R, Tolan J, Kim JS, Eriksson KL (2000). Relationships between activities of xylanases and xylan structures. *Enzyme and Microbial Technology*.

[38] Hespell RB, Cotta MA (1995). Degradation and utilization by *Butyrivibrio fibrisolvens* H17c of xylans with different chemical and physical properties. *Applied and Environmental Microbiology*.

[39] Havukainen R, Törröen A, Laitinen T, Rouvinen J (1996). Covalent binding of three epoxyalkyl xylosides to the active site of endo-1,4-xylanase II from *Trichoderma reesei*. *Biochemistry*.

[40] Sanchez-Ruiz JM (1992). Theoretical analysis of Lumry-Eyring models in differential scanning calorimetry. *Biophysical Journal*.

[41] Grasso D, La Rosa C, Milardi D, Fasone S (1995). The effects of scan rate and protein concentration on DSC thermograms of bovine superoxide dismutase. *Thermochimica Acta*.

[42] Arriaga P, Menendez M, Villacorta JM, Laynez J (1992). Differential scanning calorimetric study of the thermal unfolding of *β*- lactamase I from *Bacillus cereus*. *Biochemistry*.

[43] Cooper A, Nutley MA, Wadood A, Harding SE, Chowdry BZ (2001). Differential scanning calorimetry. *Protein-Ligand Interaction: Hydrodynamics and Calorimetry*.

[44] Freire E, Biltonen RL (1978). Thermodynamics of transfer ribonucleic acids: the effect of sodium on the thermal unfolding of yeast tRNA(Phe). *Biopolymers*.

[45] Gasset M, Saiz JL, Laynez J (1997). Conformational features and thermal stability of bovine seminal plasma protein PDC-109 oligomers and phosphorylcholine-bound complexes. *European Journal of Biochemistry*.

[46] Shental-Bechor D, Levy Y (2008). Effect of glycosylation on protein folding: a close look at thermodynamic stabilization. *Proceedings of the National Academy of Sciences of the United States of America*.

[47] Anbarasan S, Jänis J, Paloheimo M (2010). Effect of glycosylation and additional domains on the thermostability of a family 10 xylanase produced by *Thermopolyspora flexuosa*. *Applied and Environmental Microbiology*.

[48] Torronen A, Mach RL, Messner R (1992). The two major xylanases from *Trichoderma reesei*: characterization of both enzymes and genes. *Biotechnology*.

[49] Lappalainen A, Siika-Aho M, Kalkkinen N, Fagerström R, Tenkanen M (2000). Endoxylanase II from *Trichoderma reesei* has several isoforms with different isoelectric points. *Biotechnology and Applied Biochemistry*.

[50] Okada H, Wakamatsu M, Takano Y, Nogawa M, Morikawa Y (1999). Expression of two *Trichoderma reesei* xylanases in the fission yeast *Schizosaccharomyces* pombe. *Journal of Bioscience and Bioengineering*.

[51] P. Biely (1994). Stereochemistry of the hydrolysis of glycosidic linkage by endo-*β*-1,4-xylanases of *Trichoderma reesei*. *The FEBS Letters*.

[52] Gómez M, Isorna P, Rojo M, Estrada P (2001). Kinetic mechanism of *β*-xylosidase from *Trichoderma reesei* QM 9414. *Journal of Molecular Catalysis B: Enzymatic*.

[53] Lozano P, Combes D, Iborra JL (1994). Effect of polyols on α-chymotrypsin thermostability: a mechanistic analysis of the enzyme stabilization. *Journal of Biotechnology*.

[54] Wimmer R, Olsson M, Neves Petersen MTN, Hatti-Kaul R, Petersen SB, Müller N (1997). Towards a molecular level understanding of protein stabilization: the interaction between lysozyme and sorbitol. *Journal of Biotechnology*.

[55] Gekko K (1982). Calorimetric study on thermal denaturation of lysozyme in polyol-water mixtures. *Journal of Biochemistry*.

