# Supplementary material for: Effect of Temperature on Xylanase II from Trichoderma reesei QM 9414: A Calorimetric, Catalytic, and Conformational Study
Source: Enzyme Res. 2014 Sep 7;2014:708676. doi: 10.1155/2014/708676 (PMC4170777; doi:10.1155/2014/708676)
Supplement: Supplementary file 1 — Supplementary materials deal with the non-competitive inhibition of xylanase by D-gluconolactone, the inhibition constants being determined. Also, the effect of temperature on xylanase activity is presented according to the Arrhenius plot of the data, the activation and inactivation energies for the catalysis of xylan being determined. [file 708676.f1.doc]

**Effect of D-glucono-1,5 lactone (GL) on xylanase kinetics**

To establish the pattern of xylanase inhibition by GL, 0.3 M and 0.4 M GL were used as inhibitors on the xylanase kinetics with 1-12 mg·mL^-1^ beechwood xylan as substrate. and 1.3 μg protein. Data from the inhibited and non inhibited reaction were fitted by non-lineal regression toEq.:

$$v=\frac{V\cdot s}{Km\cdot s}$$

where v is activity (μmol·min^-1^·mg^-1^), s the substrate concentration (mg·mL^-1^), V the maximum velocity and Km the Michaelis constant. Data in plot are presented as double reciprocal plots (Fig 1A), in order to facilitate pattern recognition. Replots of intercepts (I=V^-1^, Fig 1B) and slopes (S=Km·V^-1^, Fig 1C) vs inhibitor concentration show that the inhibition is I-linear and S-linear. The fitting of data by linear regression gave the inhibition constants for the slope (KiS = 0.355 M) and for the intercept (KiI = 0.347 M), which are practically identical, since the lines in the double reciprocal plot (Fig 1A), cross-over in the X axis. This is an expected result as the replots are defined by the same function (actually linear) [1]. The GL inhibition is of the classical non competitive type, a particular case of mixed-inhibition occurring when the inhibitor has the same affinity for the free enzyme than for the enzyme-substrate complex, and thus KiS = KiI [2]. Nevertheless, we must exercise caution regarding enzyme inhibition since the classical interpretation of competitive inhibition has been developed with enzymes catalyzing soluble substrates, where the highest substrate concentrations can compete with the inhibitors to bind to the enzyme active site. On the contrary, insoluble substrates show limited diffusion making the competition with the inhibitors difficult and sometimes hiding the true inhibition patterns. In this context, it has been found that GL shows non-competitive inhibition on the cellulose hydrolysis by a cellulase from *T. reesei* but competitive inhibition when a soluble derivative of cellulose (carboxymethylcellulose) was employed as substrate [3]. With regard to hemicellulases, we have found that GL is a competitive inhibitor on the *T. reesei* β-xylosidase kinetics with a soluble substrate (*p*-nitrophenyl-β-D-xylopyranoside) [4]. As the effect of GL on xylanase kinetics has been studied with insoluble beechwood xylan, it could be that the non-competitive inhibition showed below would change to competitive if a soluble substrate was employed. Nevertheless, the soluble xylan we have prepared is not suitable for kinetic studies due to its high sugar content (1.15 μmol·mL^-1^) which would mask the reducing sugars produced in the enzymatic reaction. As part of those sugars would act as substrate (medium and short saccharides) and part as inhibitors (very short saccharides), the interpretation of the inhibition pattern would be very difficult and we decided not to carry out the kinetic studies with soluble xylan.

[1] J. A. Todhunter, “Reversible enzyme inhibition”, *Methods in Enzymology*, vol. 63, pp. 383-459, 1979.

[2] W. W. Cleland, “Steady state kinetics”, in P. Boyer (ed), T*he Enzymes*, Academic Press, New York, pp. 1-65, 1970.

[3] M. Hotzapple, M. Cognata, Y. Shu and C. Hendrikson, “Inhibition of *Trichoderma reesei* Cellulase by Sugars and Solvents”, *Biotechnology and Bioengineering*, vol. 36, pp. 275-287, 1990.

[4] M. Gómez, P. Isorna, M. Rojo and P. Estrada, “Kinetic mechanism of β-xylosidase from *Trichoderma reesei* QM9414”, *Journal of Molecular Catalysis B: Enzymatic*, vol. 16, pp. 7-17, 2001.

**Figure 1** A) Double recriprocal plots of xylanase without and with 0.3 and 0.4 M GL. B) Replot of intercepts vs GL concentration. C) Replot of slopes vs GL concentration. Data in B and C were fitted by linear regression.

**Effect of temperature on xylanase activity**

The Arrhenius plot of 1.3 μg xylanase when the enzymatic assays were carried out at the indicated temperatures is shown in Fig 2. The lines are the fit by linear regression of data in the 20-55 ºC (dotted line), 55-65 ºC and 65-90 ºC (solid lines) intervals to Eq.:

$$\ln v=\ln A+\frac{Ea}{RT}$$

where v is velocity (activity) A is a constant, Ea the activation energy (kJ·mol^-1^), T the absolute temperature (K) and R the gas constant (8.314 J·mol^-1^·K^-1^). The activation energy for catalysis (Ea = 32 kJ·mol^-1^) and the activation energy for the inactivation in two steps (Eai_1_ = 14.3 kJ·mol^-1^ and Eai_2_ = 119·64 kJ·mol^-1^ respectively) were obtained in the 20-55 ºC, 55-65 ºC and 65-90 ºC intervals respectively. An activation energy around 32 kJ·mol^-1^ is indicative that there is not a great energy barrier for the hydrolysis of xylan by xylanase, that is, a small increase in temperature will increase exponentially the number of activated enzyme molecules with an energy ≥ Ea, and consequently will be able to react. Above the optimum temperature for catalysis, the loss of activity occurs in two steps, reinforcing the idea that more than two states are involved in xylanase thermal unfolding.

**Figure 2** The Arrhenius plot of xylanase
